# *N*-n-Butyl Haloperidol Iodide, a Derivative of the Anti-psychotic Haloperidol, Antagonizes Hypoxia/Reoxygenation Injury by Inhibiting an Egr-1/ROS Positive Feedback Loop in H9c2 Cells

**DOI:** 10.3389/fphar.2018.00019

**Published:** 2018-01-25

**Authors:** Ting Sun, Yanmei Zhang, Shuping Zhong, Fenfei Gao, Yicun Chen, Bin Wang, Wenfeng Cai, Zhaojing Zhang, Weiqiu Li, Shishi Lu, Fuchun Zheng, Ganggang Shi

**Affiliations:** ^1^Department of Pharmacology, Shantou University Medical College, Shantou, China; ^2^Department of Biochemistry and Molecular Biology, University of Southern California, Los Angeles, CA, United States; ^3^Department of Medical Genetics and Cell Biology, School of Basic Medical Sciences, Zhengzhou University, Zhengzhou, China; ^4^Analytical Cytology Laboratory, Shantou University Medical College, Shantou, China; ^5^Department of Pharmacy, The First Affiliated Hospital, Shantou University Medical College, Shantou, China; ^6^Clinical Pharmacology Laboratory, The First Affiliated Hospital, Shantou University Medical College, Shantou, China

**Keywords:** *N*-n-butyl haloperidol, reactive oxygen species, Egr-1, SIRT1, hypoxia/reoxygenation

## Abstract

Early growth response-1 (Egr-1), a transcription factor which often underlies the molecular basis of myocardial ischemia/reperfusion (I/R) injury, and oxidative stress, is key to myocardial I/R injury. Silent information regulator of transcription 1(SIRT1) not only interacts with and is inhibited by Egr-1, but also downregulates reactive oxygen species (ROS) via the Forkhead box O1(FOXO1)/manganese superoxide dismutase (Mn-SOD) signaling pathway. *N*-n-butyl haloperidol iodide (F_2_), a new patented compound, protects the myocardium against myocardial I/R injury in various animal I/R models *in vivo* and various heart-derived cell hypoxia/reoxygenation (H/R) models *in vitro*. In addition, F_2_ can regulate the abnormal ROS/Egr-1 signaling pathway in cardiac microvascular endothelial cells (CMECs) and H9c2 cells after H/R. We studied whether there is an inverse Egr-1/ROS signaling pathway in H9c2 cells and whether the SIRT1/FOXO1/Mn-SOD signaling pathway mediates this. We verified a ROS/Egr-1 signaling loop in H9c2 cells during H/R and that F_2_ protects against myocardial H/R injury by affecting SIRT1-related signaling pathways. Knockdown of Egr-1, by siRNA interference, reduced ROS generation, and alleviated oxidative stress injury induced by H/R, as shown by upregulated mitochondrial membrane potential, increased glutathione peroxidase (GSH-px) and total SOD anti-oxidative enzyme activity, and downregulated MDA. Decreases in FOXO1 protein expression and Mn-SOD activity occurred after H/R, but could be blocked by Egr-1 siRNA. F_2_ treatment attenuated H/R-induced Egr-1 expression, ROS generation and other forms of oxidative stress injury such as MDA, and prevented H/R-induced decreases in FOXO1 and Mn-SOD activity_._ Nuclear co-localization between Egr-1 and SIRT1 was increased by H/R and decreased by either Egr-1 siRNA or F_2_. Therefore, our results suggest that Egr-1 inhibits the SIRT1/FOXO1/Mn-SOD antioxidant signaling pathway to increase ROS and perpetuate I/R injury. F_2_ inhibits induction of Egr-1 by H/R, thereby activating SIRT1/FOXO1/Mn-SOD antioxidant signaling and decreasing H/R-induced ROS, demonstrating an important mechanism by which F_2_ protects against myocardial H/R injury.

## Introduction

Ischemic heart disease is a major cause of mortality worldwide (Roger et al., 2011). Myocardial ischemia and reperfusion (I/R) injury is a common pathological physiological phenomenon in ischemic heart disease and cardiac surgery, typically arising from restoration of oxygenated blood flow after ischemia, which may lead to aggravated metabolic disorders, structural damage, and even irreversible damage (Thiel and Cibelli, 2002). However, its pathogenesis is incompletely understood. Myocardial I/R injury is a multifactorial process in which oxidative stress, inflammation, and intracellular Ca^2+^ overload contribute. Early growth response protein 1 (Egr-1), a transcription factor, has been found to play a role in multiple pathways, such as differentiation, proliferation, and apoptosis (Shin et al., 2010; Zwang et al., 2012). Many studies suggest that Egr-1 is a master switch for various pathways of reperfusion injury because its overexpression is closely related to I/R injury (Yan et al., 2000; Frank et al., 2012; Wu et al., 2013).

Numerous studies have shown that reactive oxygen species (ROS)-induced oxidative stress is key to myocardial I/R injury (Murphy and Steenbergen, 2008; Radak et al., 2011; Minamino, 2012). Both Egr-1 and ROS are closely related to I/R injury, and our previous research has shown that the ROS/MAPK/Egr-1 signaling pathway is activated during hypoxia/reoxygenation (H/R) models of myocardial and cardiac microvascular endothelial cells (CMEC) (Zhang et al., 2015; Lu S. et al., 2016). Bek’s group reported that over-expressed Egr-1 in proximal tubule epithelial cells downregulates expression of Cu/Zn-SOD and Mn-SOD, resulting in increased tissue ROS (Bek et al., 2003). Meanwhile, our previous work shows that oxidative stress damage, as assessed by SOD activity and MDA, could be alleviated by expression of an Egr-1 antisense oligonucleotide within *in vivo* and *in vitro* I/R models (Zhang et al., 2007). Thus, Egr-1 may affect ROS in the myocardium by regulating SOD activity or by other mechanisms. Therefore, we speculated an inverse activation of the Egr-1/ROS signaling pathway during myocardial I/R via a ROS/Egr-1 signaling loop between Egr-1 and ROS in myocardial I/R injury based on former study results.

*N*-butyl haloperidol iodide (F_2_), a novel compound derived from haloperidol, has been shown to antagonize myocardial I/R injury by reducing infarct size and leakage of various enzymes, improving cardiac function, and reducing oxidative stress in various animal models *in vivo* and various heart-derived cells models *in vitro* (Gao et al., 2004, 2010; Zhou et al., 2004; Zhang et al., 2006; Lu B. et al., 2016). The protective mechanism of F_2_ might be associated with blocking L-type calcium channels and suppressing the overexpression of Egr-1. If there is an ROS/Egr-1 positive feedback loop in myocardial I/R, the protective effect of F_2_ should be related to regulation of this ROS/Egr-1 loop.

Silent information regulator of transcription 1 (SIRT1) is sirtuin family member of class III histone deacetylases, which depends on nicotinamide adenine dinucleotide (NAD^+^) (Haigis and Sinclair, 2010; Hsu et al., 2010). Many studies show that activation of SIRT1 protects against I/R injury (Brunet et al., 2004; Tanno et al., 2010; Lempiainen et al., 2012; Shin et al., 2012). SIRT1 activates Forkhead box O1 (FOXO1) by deacetylation of acetylated FOXO1 (Ac-FOXO1), which upregulates expression of antioxidant enzymes, such as manganese superoxide dismutase (Mn-SOD) and glutathione peroxidase (GSH-px), decreases ROS and resists oxidative stress (Daitoku et al., 2004; Tong et al., 2013). Egr-1 can induce expression of SIRT1 by activating the SIRT1 promoter. However, a recent study in skeletal muscle cells shows that Egr-1 can physically interact with and inhibit the activity of SIRT1 (Pardo and Boriek, 2012). Therefore, we assumed that overexpressed Egr-1 may affect ROS by regulating SIRT1 in myocardium subjected to I/R, and the mechanism of overexpressed Egr-1 on the antioxidant activity of SIRT has been our research focus: (1) whether the activity of SIRT1 increases because Egr-1 activates SIRT1’s promoter when myocardium is suffering from I/R; (2) whether the activity of SIRT1 decreases because overexpressed Egr-1 directly binds to SIRT1 protein. Taken together, we studied whether there is an Egr-1/ROS signaling pathway in H9c2 cells after H/R, and whether SIRT1-related signaling (SIRT1/FOXO1/Mn-SOD) is involved in this pathway. Besides, we explored whether F_2_, which inhibits Egr-1, reduces H/R-induced cardiomyocyte injury by regulating SIRT1/FOXO1/Mn-SOD signaling pathway.

## Materials and Methods

### Reagent Preparation

SiRNAs were purchased from Shanghai Genepharma Co., Ltd. (China). Lipofectamine 2000 was purchased from Invitrogen (United States), Opti-MEM media was purchased from Life Technologies (United States). 2′,7′-Dichlorofluorescein acetyl acetate (DCFH-DA) was purchased from Sigma–Aldrich (United States). F_2_ was synthesized in our laboratory and dissolved in DMSO (≤0.1%). The following primary antibodies were purchased from Cell Signaling Technology (United States): rabbit anti-Egr-1, mouse anti-SIRT1, and rabbit anti-FOXO1 antibody. Rabbit anti-Ac-FOXO1 antibody was purchased from Santa Cruz Biotechnology (United States). Mouse β-actin antibody, anti-rabbit secondary antibodies and anti-mouse secondary antibodies were purchased from Wuhan Boster Biotechnology Limited Company (China). Alexa Fluor 488 goat anti-mouse IgG and Alexa Fluor 594 goat anti-rabbit IgG were purchased from Life Technologies (United States). All reagent kits for real-time RT-PCR were purchased from TaKaRa Biotechnology (China). JC-1 was purchased from Beyotime Biotechnology (China).

### H9c2 Cell Culture and H/R Protocol

The H9c2 cell line was purchased from the American Type Culture Collection and cultured in DMEM (Gibco, United States) supplemented with 10% fetal bovine serum (Biowest, France) at 37°C with 5% CO_2_. To induce hypoxia, H9c2 cells were cultured in hypoxic solution (137 mM NaCl, 12 mM KCl, 0.49 mM MgCl_2_⋅6H_2_O, 0.9 mM CaCl_2_, 4 mM HEPES, and 20 mM sodium lactate) and placed in an air-tight chamber gassed with pure N_2_ for 2, 4, 6, or 8 h at 37°C, after which the hypoxia solution was then replaced with fresh oxygenated culture medium, and the culture vessels were transferred to a normoxic incubator (5% CO_2_) at 37°C for 1 h of reoxygenation. F_2_ (1 × 10^-6^ M) was prepared in normal medium (pre-incubated 30 min), hypoxia solution, and/or reoxygenation medium (F_2_+H/R group).

### Egr-1 Small Interfering RNA (siRNA)

Cells were cultured in 24-well plates and transfected with Egr-1-siRNA using Lipofectamine 2000. First, 3.75 μL of Egr-1-siRNA (20 μM) was mixed with Opti-MEM media, and Lipofectamine 2000 was mixed with Opti-MEM in another Eppendorf tube, and then the mixtures were combined for 20 min at 25°C. Then, the mixture was added to culture plates for 6 h, after which medium was changed to antibiotic-free DMEM supplemented with 10% FBS for 48 h. After that, H/R was applied. There were nine experimental groups: control, H/R, negative control+H/R (NC+H/R), Egr-1-siRNA (siRNAs 1–6) +H/R (siRNA+H/R).

Egr1-siRNA-1: Sense, 5′-CAGACAUGACAGCAACCUUd TdT-3′, Anti-sense, 5′-AAGGUUGCUGUCAUGUCUGdTdT-3′

Egr1-siRNA-2: Sense, 5′-CUCACUCCACUAUCCACUAd TdT-3′, Anti-sense, 5′-UAGUGGAUAGUGGAGUGAGdTdT-3′

Egr1-siRNA-3: Sense, 5′-CCAAGGGUGGUUUCCAGGUd TdT-3′, Anti-sense, 5′-ACCUGGAAACCACCCUUGGdTdT-3′

Egr1-siRNA-4: Sense, 5′-CCAGGACUUAAAGGCUCUUd TdT-3′, anti-sense, 5′-AAGAGCCUUUAAGUCCUGGdTdT-3′

Egr1-siRNA-5: Sense, 5′-CCAGGAGUGAUGAACGCAAd TdT-3′, Anti-sense, 5′-UUGCGUUCAUCACUCCUGGdTdT-3′

Egr1-siRNA-6: Sense, 5′-CAACGACAGCAGUCCCAUUd TdT-3′, Anti-sense, 5′-AAUGGGACUGCUGUCGUUGdTdT-3′

Negative control: Sense, CGUUUGUUCGCUUCCUGAGTT, Anti-sense, CUCAGGAAGCGAACAAACGTG.

### Quantitative Real-Time PCR (RT-PCR)

Egr-1 gene expression was assayed by RT-PCR. Total RNA from H9c2 cells was extracted with Trizol reagent. A PrimeScript RT reagent kit was used to synthesize first-strand cDNA. Then cDNAs were quantified by real-time PCR on an ABI 7500 Real-Time PCR System (Applied Biosystems). Target mRNA expression was normalized to GAPDH mRNA. Primers were synthesized by HuaDa Gene Technology and are shown below:

Egr-1:

F5′-GAACAACCCTACGAGCACCTG-3′;

R5′-GCCACAAAGTGTTGCCACTG-3′;

GADPH:

F5′-GGCACAGTCAAGGCTGAGAATG-3′;

R5′-ATGGTGGTGAAGACGCCAGTA-3′.

### Western Blotting

H9c2 cells were washed with cold PBS three times, and scraped into mixed lysate buffer containing RIPA buffer and phenylmethanesulfonyl fluoride (PMSF; 1,000:1). Lysates were centrifuged for 15 min at 12,000 × *g* at 4°C. Protein was quantified using a BCA protein assay kit (Pierce, Rockford, IL, United States), and 25 μg of each sample was resolved on 10% SDS-PAGE (stacking gel 50 V, separating gel 100 V), and transferred to nitrocellulose membranes (100 V, 75 min). Then, membranes were blocked with 5% fat-free milk, and then incubated overnight at 4°C with primary antibody (Egr-1—1: 1,000, SIRT1—1: 1,000, Ac-FOXO1—1:200, FOXO1—1: 1,000 and β-actin—1: 3,000), followed by incubation with HRP-conjugated goat anti-rabbit IgG (1: 20,000) or HRP-labeled goat anti-mouse IgG (1: 30,000) for 1 h at room temperature. Protein bands were analyzed with Gel-pro Image Analysis Software (Media cybernetics, United States).

### Intracellular ROS Using Flow Cytometry

Reactive oxygen species in H9c2 cells were measured using a peroxide-sensitive fluorescent probe DCFH-DA and flow cytometry. Briefly, cells were incubated with 10 μM DCFH-DA for 30 min at 37°C in the dark. Then, cells were washed three times with PBS, and monitored with flow cytometry 488 nm_ex_ and 525 nm_em_. Data are a fluorescent ratio of each group to controls. The mean fluorescent intensity (MFI) was analyzed on a BD Accuri C6 flow cytometer (United States).

### Measurement of Mitochondrial Membrane Potential

When the mitochondrial membrane potential (MMP) is high, such as in normal cells, JC–1 concentrates in the mitochondrial matrix, forming J-aggregates, which produces a red fluorescence. In contrast, when MMP is low, after H/R stimulation, JC-1 is retained in the cytoplasm as a monomer, unable to concentrate within the mitochondrial matrix, resulting in green fluorescence. Cells were incubated with JC-1 for 20 min at 37°C in the dark, and images were taken with a fluorescent microscope (Olympus, Japan).

### Measurement of MDA and Mn-SOD and GSH-px Activity

Intracellular of MDA, total SOD, CuZn-SOD and GSH-px activity in H9c2 cells measured using a colorimetric assay kit (Jiancheng Bioengineering Institute, Nanjing, China) according to the manufacturer’s instructions. Mn-SOD activity = total SOD activity-CuZn-SOD activity. All results were expressed as units/mg protein.

### Co-localization of Egr-1 and SIRT1

H9c2 cells were inoculated onto sterile cover glasses placed in each well of a 24-well dish. After treatment, cells were washed twice with cold PBS, and fixed in 4% paraformaldehyde for 20 min at room temperature. After permeabilization with 0.3% Triton X-100, cells were washed twice with cold PBS and blocked with goat serum for 1 h at room temperature. Primary rabbit anti-Egr-1(1:50) and mouse anti-SIRT1 (1:100) antibodies were incubated with cells overnight at 4°C, followed by incubation for 1 h in the dark with secondary antibodies (Alexa Fluor 594-conjugated goat anti-rabbit IgG or Alexa Fluor 488-conjugated goat anti-mouse IgG). Then cell nuclei were stained with Hoechst 33258 (Beyotime Biotechnology, China) for 15 min at room temperature. Finally, the cover glasses were mounted on a glass slide, and images were observed with a confocal laser scanning microscope (Olympus, Tokyo, Japan).

### Statistical Analysis

Data are shown as mean ± SEM. Comparisons between groups were analyzed by one-way ANOVA followed by a Student–Newman–Keuls test (*p* < 0.05 was considered statistically significant).

## Results

### ROS, MDA, and Egr-1 Protein Expression in H9c2 Cells after H/R

**FIGURE 1:**
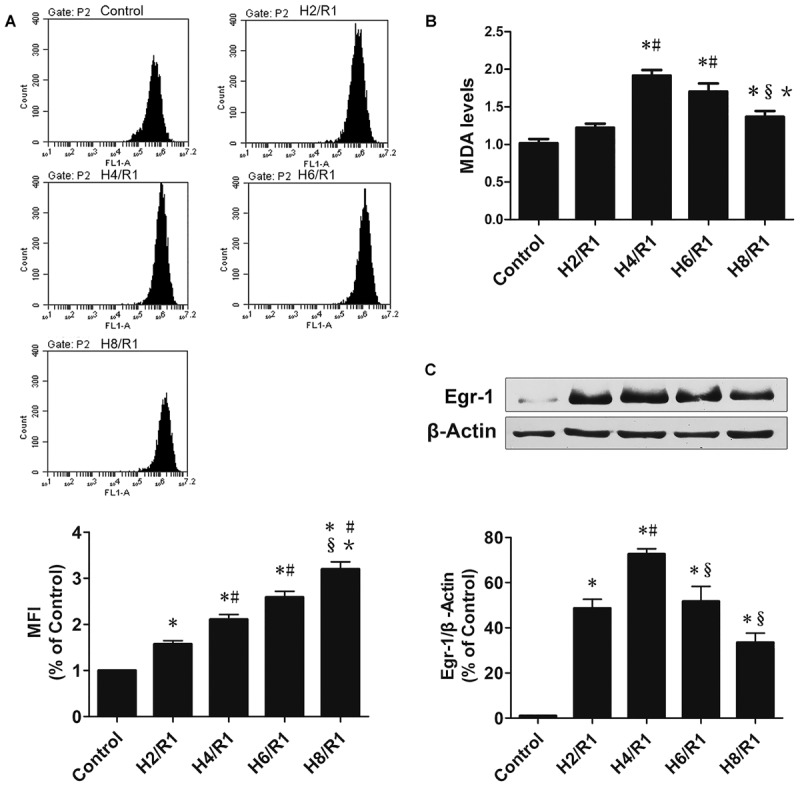
Reactive oxygen species (ROS) and MDAs and Egr-1 protein expression in H9c2 cells at various times following H/R. **(A)** ROS s determined by flow cytometry, *n* = 3. **(B)** Level of MDA, *n* = 6. **(C)** Representative Egr-1 western blot and quantitation of Egr-1 protein expression by western blotting analysis, *n* = 3. All values are means ± SEM.; ^∗^*p* < 0.05 vs. control group; ^#^*p* < 0.05 vs. H2/R1 group; ^x^*p* < 0.05 vs. H4R1 group; ^∗^*p* < 0.05 vs. H6/R1 group.

H/R increased ROS and **Figure 1** depicts the hourly data for reperfusion groups (**Figure 1A**). Compared with controls, MDA increased at each time point, but not significantly in the H2/R1 group. In addition, MDA peaked occurred at H4/R1 (**Figure 1B**). We measured Egr-1 protein expression in H9c2 cells with Western blot. As shown in **Figure 1C**, compared with controls, Egr-1 protein expression increased in all H/R groups, and peaked in the H4/R1 group, as well. Thus, 4 h of hypoxia and 1 h of reoxygenation were used in subsequent experiments.

### Effects of Egr-1 siRNA and F_2_ on Egr-1 Expression in H9c2 Cells

**FIGURE 2:**
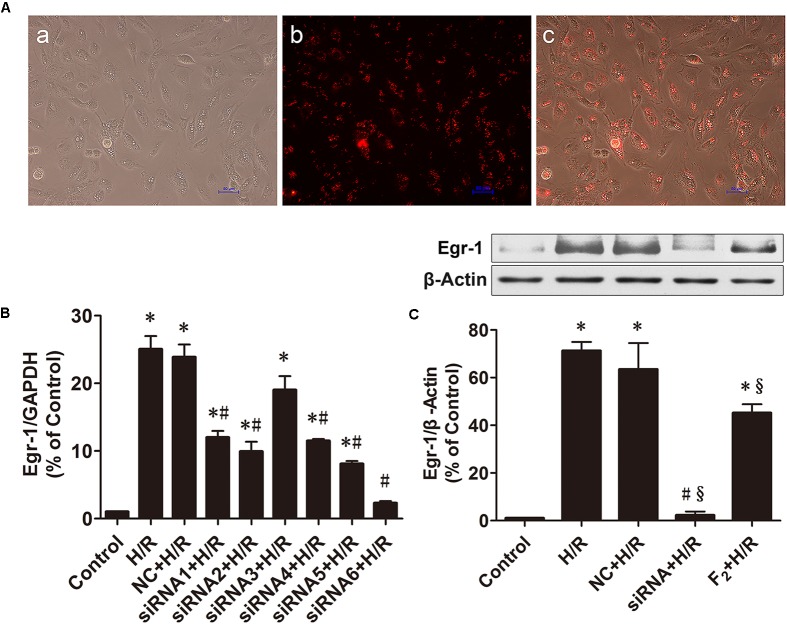
Effects of Egr-1 siRNA and F_2_ on expression of Egr-1 in H9c2 cells. **(A)** Transfection efficiency of siRNA observed under an inverted fluorescent microscope, (a) bright field, (b) red fluorescence reflects cells transfected with negative control siRNA, (c) merged image (bar = 50 μm). **(B)** QRT-PCR of Egr-1 mRNA expression. **(C)** Western blot of Egr-1 protein expression. All values are means ± SEM., *n* = 3; ^∗^*p* < 0.05 vs. control group; ^#^*p* < 0.05 vs. NC+H/R group; ^x^*p* < 0.05 vs. H/R group.

SiRNA labeled with fluorescent dye Cy3 was transfected into H9c2 cells and transfection efficiency was observed under an inverted fluorescent microscope [**Figure 2A**, (a) bright field, (b) red fluorescence reflects siRNA negative control, (c) a and b merged]. The proportion of fluorescent cells was >90%, indicating transfection efficiency >90%. As shown in **Figure 2B**, all Egr-1 siRNAs (siRNA1-siRNA6) decreased H/R-mediated induction of Egr-1 mRNA in H9c2 cells. Egr-1 siRNA6 maximally reduced Egr-1 expression. Thus, subsequent experiments used Egr-1 siRNA6. Expression of Egr-1 protein data were similar. H/R upregulated Egr-1 protein expression, and Egr-1 siRNA blocked this change. In addition, F_2_ attenuated H/R-induced increases in Egr-1 protein expression (**Figure 2C**).

### Effect of Egr-1 siRNA and F_2_ on ROS s in H9c2 Cells during H/R

**FIGURE 3:**
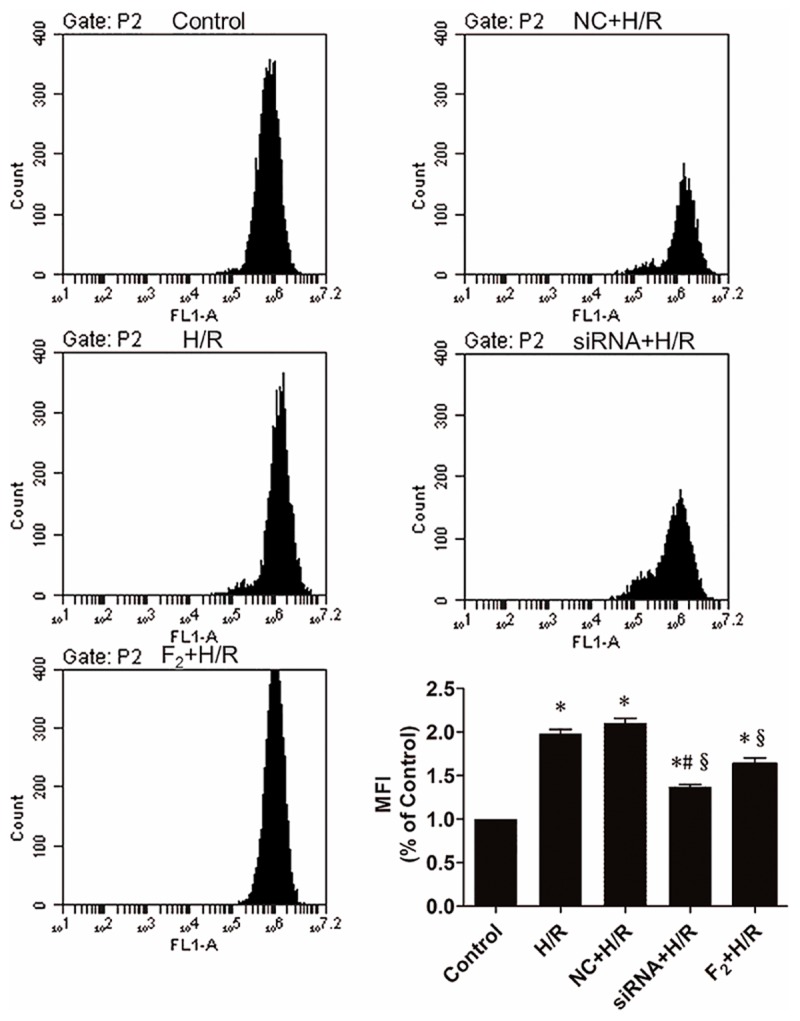
Reactive oxygen species in H9c2 cells as measured by flow cytometry. All values are means ± SEM., *n* = 5; ^∗^*p* < 0.05 vs. controls; ^#^*p* < 0.05 vs. the NC+H/R group; ^x^*p* < 0.05 vs. the H/R group.

Reactive oxygen species production was significantly increased in H/R and NC+H/R groups compared with controls (**Figure 3**). Nonetheless, compared with the NC+H/R group, Egr-1 siRNA downregulated ROS significantly and F_2_ treatment decreased production of ROS induced by H/R.

### Egr-1 siRNA and F_2_ Attenuate H/R-Induced Decreases in Mitochondrial Membrane Potential in H9c2 Cells

**FIGURE 4:**
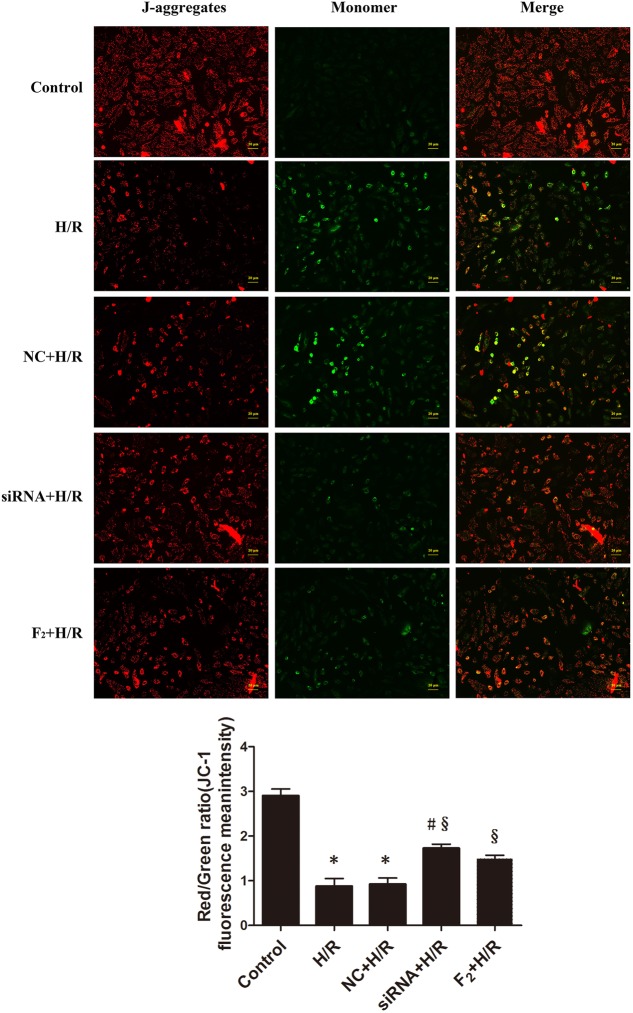
H/R-mediated reduction of mitochondrial membrane potential is reduced by Egr-1 inhibition or treatment with F_2_ in H9c2 cells. H9c2 cells, treated with Egr-1 siRNA or F_2_, underwent H/R and were stained with JC-1. High-intensity red fluorescence reflected higher membrane potential and high-intensity green fluorescence indicated lower membrane potential (bar = 20 μm). Quantitative data were the ratio of red to green. All values are means ± SEM, *n* = 3; ^∗^*p* < 0.05 vs. control group; ^#^*p* < 0.05 vs. NC+H/R group; ^x^*p* < 0.05 vs. H/R group.

Mitochondria act as a nexus for reperfusion injury pathways, and loss of mitochondrial transmembrane potential indicates mitochondrial dysfunction (Mailloux et al., 2013; Patil et al., 2015). Mitochondrial membrane potential used to assess the degree of mitochondria damage was assessed after incubating cells with JC-1, and high-intensity, punctate red fluorescence reflected more membrane potential. Diffuse, high-intensity green fluorescence reflected lower membrane potential. Compared with controls, H/R reduced mitochondrial membrane potential. However, Egr-1 siRNA inhibited H/R-induced reduction in mitochondrial membrane potential. Likewise, F_2_ treatment increased mitochondrial membrane potential. Thus, Egr-1 siRNA and F_2_ alleviated the degree of mitochondria damage (**Figure 4**).

### Effects of Egr-1 siRNA and F_2_ on Level/Activity of MDA, GSH-px, Total SOD and Mn-SOD in H9c2 Cells Stimulated by H/R

Intracellular level/activity of MDA, GSH-px, total SOD and Mn-SOD were measured to assess the degree of oxidative stress injury and anti-oxidative ability in H9c2 cells during H/R. **Table 1** shows that H/R significantly increased MDA, and Egr-1 siRNA and F_2_ markedly decreased this change. Compared with controls, activity GSH-px, total SOD and Mn-SOD were decreased by H/R, but increased by Egr-1 siRNA and F_2_.

**Table 1:** MDA, GSH-px and Mn-SOD in H9c2 cells (mean ± SEM, *n* = 8).

Group	MDA (nmol/mg prot)	GSH-px (U/mg prot)	Total SOD (U/mg prot)	Mn-SOD (U/mg prot)
Control	1.07 ± 0.08	27.25 ± 1.14	32.55 ± 1.94	6.22 ± 0.46
H/R	2.18 ± 0.16*	15.70 ± 0.84*	20.12 ± 1.38*	0.76 ± 0.15*
NC+H/R	2.21 ± 0.16*	15.96 ± 0.57*	18.75 ± 0.95*	0.80 ± 0.20*
siRNA+H/R	1.44 ± 0.10#§	21.36 ± 0.72*#§	28.51 ± 1.63#§	4.40 ± 0.29#§
F_2_+H/R	1.59 ± 0.13§	19.38 ± 1.08*§	28.46 ± 1.29§	5.47 ± 0.33§


*^∗^*P* < 0.05 vs. control group; ^#^*P* < 0.05 vs. NC+H/R group; ^x^*P* < 0.05 vs. H/R group.*

### Effects of Egr-1 siRNA and F_2_ on SIRT1, Ac-FOXO1, and FOXO1 Expression in H9c2 Cells Subjected to H/R

**FIGURE 5:**
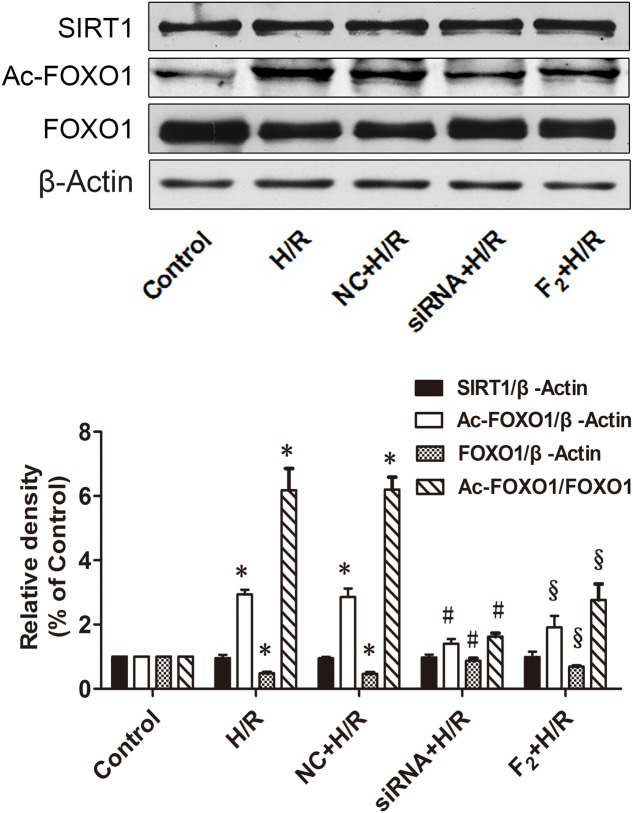
Effects of Egr-1 siRNA and F_2_ on SIRT1, Ac-FOXO1, and FOXO1 expression in H9c2 cells subjected to H/R. Representative bands and quantitative analysis results of SIRT1, Ac-FOXO1, and FOXO1. Quantitative densitometric data are expressed as percents of controls. All values are means ± SEM., *n* = 3; ^∗^*p* < 0.05 vs. control group; ^#^*p* < 0.05 vs. NC+H/R group; ^x^*p* < 0.05 vs. H/R group.

As shown in **Figure 5**, there was no significant difference in SIRT1 protein expression among all groups. FOXO1 expression decreased but Ac-FOXO1 expression and the radio of Ac-FOXO1/FOXO1 increased significantly in the H/R group. Pre-treatment with either Egr-1 siRNA or F_2_ significantly increased FOXO1 expression, decreased Ac-FOXO1 expression and the ratio of Ac-FOXO1/FOXO1. Thus, although H/R had no effect on expression of SIRT1, H/R did inhibit the activity of SIRT1, as evidenced by decreased deacetylation of Ac-FOXO1 (increase ratios of Ac-FOXO1/FOXO1), and the decrease could be blocked by either Egr-1 siRNA or F_2_.

### Effect of Egr-1 siRNA and F_2_ on Expression and Co-location of Egr-1 and SIRT1 in H9c2 Cells after H/R

**FIGURE 6:**
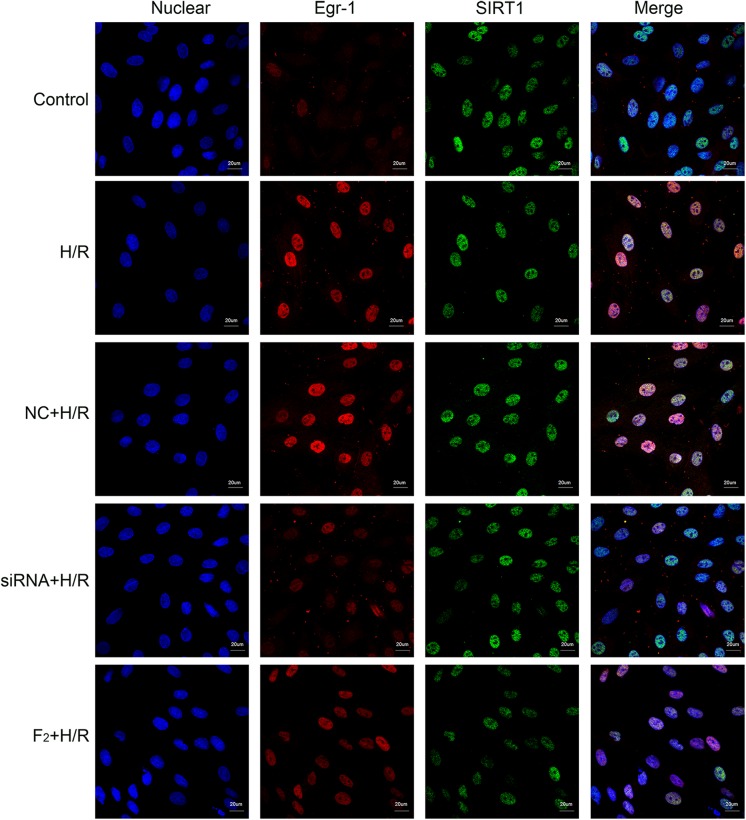
Effect of Egr-1 siRNA and F_2_ on the expression and co-localization of Egr-1 and SIRT1 in H9c2 cells observed with confocal laser scanning microscopy. H9c2 cells, treated with Egr-1 siRNA or F_2_, underwent H/R followed by immunofluorescent analysis (bar = 20 μm). Blue fluorescence reflects nuclear localization; red fluorescence reflects Egr-1 protein localization; and green fluorescence reflects SIRT1 protein localization. In controls, red fluorescence was distributed throughout the nucleus and cytoplasm. In the H/R group, red fluorescent intensity increased and was focused in the nuclei. In the NC+H/R group, red fluorescent intensity was equivalent as the H/R group. In the siRNA+H/R and F_2_ groups, red fluorescent intensity weakened compared with the NC+H/R and H/R groups. There were no marked differences in the intensity of green fluorescence among all groups.

To understand interactions between Egr-1 and SIRT1, immunofluorescent analysis was used to identify Egr-1 and SIRT1 locations. Egr-1 was distributed throughout the cell in controls and H/R caused localization of Egr-1 to the nucleus chiefly. H/R-induced nuclear localization was prevented by either siRNA-mediated Egr-1 knockdown or F_2_ treatment, consistent with results obtained by Western blot. SIRT1 was mainly in the nucleus and there were no marked differences in the intensity of green fluorescence among all groups, suggesting SIRT1 expression did not change significantly (**Figure 6**). Co-localization of Egr-1 with SIRT1 was examined using immunofluorescence and compared with controls, H/R induced Egr-1 co-localization with SIRT1 but this was inhibited by Egr-1 siRNA and F_2_ (**Figure 6**).

## Discussion

Myocardial I/R injury is an extremely complex and common pathophysiological phenomenon. Egr-1 is associated with pathological changes in I/R injury and is thought to be a “a master switch” for I/R injury because of its regulation of inflammation, coagulation, and vascular hyper-permeability which are thought to be central to pathogenesis of I/R injury (Yan et al., 2000; Zins et al., 2014; Sandoval et al., 2016). Oxidative stress also be pivotal to the pathogenesis of I/R injury. However, it is unclear whether Egr-1 can regulate oxidative stress caused by I/R stimulation. Recently, our studies on *in vitro* H/R treatment of H9c2 cells and CMECs found that H/R causes ROS generation and over-expression of Egr-1, and that ROS is upstream of Egr-1 (Zhang et al., 2015; Lu S. et al., 2016). We report that ROS generation induced by H/R is also significantly inhibited by downregulation of Egr-1, indicating that Egr-1 is upstream of ROS. Combined with previous data, these results suggest that there is a self-sustaining damaging signaling loop involving positive feedback loop between ROS and Egr-1 during myocardial I/R.

Interestingly, when examined at different H/R durations, expression of Egr-1 peaks at 4 h hypoxia and 1 h reoxygenation, whereas ROS increased continually with hypoxia. Thus, asynchronous changes may be attributed to regulation of Egr-1 which is an immediate early gene (IEG) family member that is rapidly activated by external stimuli, and then degraded. Egr-1 protein decreases after at 6 and 8 h of hypoxia, during which time the degradation of Egr-1 becomes greater than synthesis. So, overall Egr-1 is gradually decreased, but remains greater than normal, and is still sufficient for enhancing ROS, although other mechanisms may influence on ROS production. In addition, MDA increased, and then decreased. Thus, continuing cell injury explains increases in ROS and MDA decreases at 6 and 8 h of hypoxia. The longer the duration of hypoxia, the greater the cell damage. MDA easily leaks into the extracellular space when the cell lipid membrane is structurally damaged by oxidation. MDA measured was intracellular, but must have leaked into the culture medium and was not measured. However, results from other laboratories suggest that MDA content in the supernatant is higher after H/R (Pan et al., 2003; Zheng et al., 2003). Although some studies suggested that increased ROS occurs mainly in the reperfusion phase, according to our study, hypoxia time also determine the degree of oxidative stress in cells when re-oxygenation duration is unchanged. Data show that I/R relevant diseases must quickly reduce duration of hypoxia to relieve oxidative stress.

Many studies suggest that SIRT1 regulates cell proliferation, apoptosis, differentiation, senescence and metabolism (Simic et al., 2013; Chen et al., 2016; Jablonska et al., 2016), and is key to protection against ischemia by deacetylation (Hariharan et al., 2010; Shin et al., 2010; Hernandez-Jimenez et al., 2013). In addition, activation of SIRT1 reduces oxidative stress by regulating the acetylation of FOXO1 in the heart (Brunet et al., 2004; Chen et al., 2009; Chung et al., 2010) and activating antioxidant enzymes, such as Mn-SOD, catalase and GSH-px (Alcendor et al., 2007; Shalini et al., 2012). SIRT1 protects against I/R injury, but I/R-induced changes in SIRT1 expression and activity are controversial. Yu et al. (2014) reported that SIRT1 protein expression decreases in myocardial tissue at ischemia 30 min and reperfusion 6 h, and Hsu et al. (2010) reported SIRT1 protein expression and mRNA decreased at 20 min ischemia and reperfusion for 24 h. Our results show that SIRT1 protein expression remained unchanged after 4 h of hypoxia/ischemia and 1 h reoxygenation in H9c2 cells but activity decreased. This is similar to data from Liu’s group who found no significant changes in SIRT1 protein expression, but a significant reduction in activity in H9c2 cells after hypoxia for 4 h and reoxygenation for 4 h (Liu et al., 2014). Differences in outcomes may explained by differing degrees of stimulation in the models or different durations of I/R (H/R) which can change protein expression. SIRT1 may be depleted in the short time after initiation of ischemia/hypoxia (20–30 min in the above study) (Hsu et al., 2010; Yu et al., 2014), but as the duration of hypoxia progresses, Egr-1 accumulates and peaks after hypoxia for 4 h, after which re-expression of SIRT1 occurs by activating its promoter. Meanwhile, increased Egr-1 and SIRT1 leads increases the chance of interaction between the two to decrease deacetylation of SIRT1 and increase acetylated FOXO1, resulting in reduced antioxidant activity. In skeletal muscle cells, Pardo and Boriek (2012) described an automatic adjustment cycle between Egr-1 and SIRT1. Overexpression of Egr-1 protein can bind to the SIRT1 promoter and activate SIRT1 protein expression, and increased SIRT1 can then combine with Egr-1, and in turn, decrease SIRT1-dependent antioxidant enzyme expression. To verify a potential interaction between SIRT1 and Egr-1 in H9c2 cells after H/R, immunofluorescence co-localization was used to observe protein distribution and fluorescent intensity. Egr-1 was distributed throughout cell and SIRT1 was mainly localized to the nucleus of untreated cells. After H/R, Egr-1 expression increased and localized to the nucleus to co-localize with SIRT1. Egr-1 siRNA decreased the fluorescent intensity of Egr-1 staining, suggesting decreased interaction between Egr-1 and SIRT1, which would contribute to increases in expression of FOXO1 and activity of Mn-SOD. We previously found that JNK plays an important role in the ROS/Egr-1 signaling pathway in H9c2 cells and CMECs (Zhang et al., 2015; Lu S. et al., 2016). Parra et al. (2013) demonstrated that Egr-1 modulates activation of JNK-1 by siRNA technology, and Win et al. (2016) found that p-JNK increased mitochondrial ROS production in isolated mitochondria. Thus, it is worth studying whether JNK mediates the Egr1/ROS pathway in H/R model, in addition to SIRT1.

In *in vivo* and *in vitro* studies, F_2_ has been shown to have multiple protective effects on myocardial I/R injury, perhaps by suppressing overexpression of Egr-1 (Zhang et al., 2006, 2007). Moreover, F_2_ increases activity of SOD and decreases MDA in H9c2 cells and microvascular endothelial cells, after H/R, indicating that F_2_ can reduce oxidative stress injury (Lu S. et al., 2016). We report that F_2_ inhibits H/R-induced Egr-1 protein overexpression, thereby decreasing the ability of Egr-1 to inhibit SIRT1, and concomitantly enabling cells to maintain SIRT1 activity, as assessed by high Ac-FOXO1 maintained after H/R, thereby maintaining Mn-SOD activity, to reduce intracellular ROS and alleviate oxidative stress injury.

Thus, we suggest a positive feedback signaling loop between Egr-1 and ROS in H9c2 cells after H/R and an interaction pathway between oxidative stress injury and inflammatory response after H/R stimulation. Thus, therapy targeting ROS or Egr-1 may improve I/R (or H/R)-induced oxidative stress injury and the inflammatory response. Thus, F_2_ could protects cardiomyocytes from I/R or H/R injury via inhibition of the Egr-1/ROS positive feedback loop, which might extend its use in oxidative stress-related diseases such as cardiac hypertrophy.

## Conclusion

F_2_ alleviates H/R injury in H9c2 cells by blocking Egr-1-mediated inhibition of the SIRT1/FOXO1/Mn-SOD signaling pathway responsible for ROS inactivation. Based on earlier study results showing that F_2_ protects against H/R injury by inhibiting ROS/Egr-1 signaling pathways in H9c2 cells and CMECs, we conclude that the protective effect of F_2_ on the I/R myocardium is related to arresting a positive feedback signaling loop between Egr-1 and ROS.

## Author Contributions

GS and FZ supervised the overall project and helped writing the paper. TS performed the experiment and drafted the manuscript. YZ designed the experiment and analyzed the data. SZ polished the English to improve the quality of this manuscript. FG, YC, and WL contributed to figure handling or the statistical analysis. BW, WC, ZZ, and SL provided support for the experiment.

## Conflict of Interest Statement

The authors declare that the research was conducted in the absence of any commercial or financial relationships that could be construed as a potential conflict of interest.
